# Depression severity and chronic disease risk: interactive effects on cognitive function and life satisfaction in Chinese middle-aged and older adults

**DOI:** 10.3389/fpubh.2025.1530513

**Published:** 2025-06-24

**Authors:** Bowen Li, Siyu Guan, Chuan Huang, Chen Li, Yiran Du, Chunxiao Wan

**Affiliations:** Tianjin Medical University General Hospital, Department of Physical and Rehabilitation Medicine, Tianjin, China

**Keywords:** depression, cardiovascular disease, mental disorders, stroke, life satisfaction, cognitive ability

## Abstract

**Introduction:**

Mental health problems, particularly depression, are intricately linked to chronic diseases and significantly impact overall health outcomes. This study aims to investigate the dose-response relationship between depression severity and risks of heart disease, stroke, and other mental disorders, and to examine the interactive effects of depression and chronic conditions (hypertension and diabetes) on cognitive function, life satisfaction, and episodic memory among Chinese adults aged 45 and above.

**Methods:**

Using data from the China Health and Retirement Longitudinal Study (CHARLS), 13,930 participants aged 45 and above were analyzed across five waves from 2011 to 2020. Depression was measured with the CES-D-10 scale, and participants were grouped based on symptom severity. Logistic regression models were used to examine disease risks, while linear regression models assessed the interactive effects on cognitive outcomes.

**Results:**

Logistic regression models showed that higher levels of depression were significantly associated with increased risk of heart disease, while showing more complex relationships with stroke and mental illness risks. Subgroup analysis using linear regression indicated that coexisting conditions like hypertension and diabetes intensified the negative impact of depression on cognitive abilities, life satisfaction, and episodic memory.

**Discussion:**

These findings underscore the necessity of integrating mental health management into rehabilitation strategies for patients with chronic illnesses. The study suggests that addressing mental health, particularly among high-risk older adult populations with chronic diseases, may improve recovery outcomes, reduce rehabilitation duration, and enhance quality of life based on the observed associations. This highlights the importance of comprehensive care that combines mental and physical health interventions to optimize rehabilitation outcomes.

## Introduction

Mental health problems, particularly depression, are not only a major global public health challenge, but are also intricately linked to the onset and progression of various chronic diseases, including cardiovascular conditions such as heart disease and stroke, and other psychiatric disorders (1). The bidirectional relationship between depression and chronic diseases has become an increasingly important focus in public health research, as evidence accumulates regarding their complex interactions and combined effects on overall health and wellbeing (2, 3).

Depression has been consistently shown to have a direct effect not only on the occurrence but also on the course and progression of chronic diseases. Numerous studies have demonstrated that depression can worsen disease prognosis, increase mortality rates, and complicate treatment outcomes in patients with cardiovascular diseases, diabetes, and other chronic conditions (3–5). While some research suggests depression may act primarily through behavioral pathways (medication non-adherence, unhealthy lifestyle choices), others emphasize biological mechanisms including dysregulation of the hypothalamic–pituitary–adrenal axis, increased inflammation, and autonomic dysfunction (2, 6). Despite some methodological variations across studies, there is growing consensus that depression significantly impacts both the development and management of chronic diseases.

From a biological perspective, depression has been associated with systemic inflammation and endothelial dysfunction, which are key biological mechanisms contributing to cardiovascular disease development (6, 7). These physiological changes create a foundation for understanding how depression might influence chronic disease outcomes through direct biological pathways. Individuals with depression often experience diminished quality of life, heightened disease burden, and greater challenges in clinical management. Understanding how depression interacts with chronic diseases and impacts various health outcomes is essential for developing integrated prevention and intervention strategies.

The interaction between depression and chronic diseases may extend beyond direct physiological effects to indirectly influence cognitive function, life satisfaction, and episodic memory through complex pathways involving neuroendocrine dysregulation, inflammation, and brain structure alterations (7–9). This indirect effect pathway represents a critical yet understudied dimension of depression’s impact on overall health and functioning. Numerous studies have shown that older adults with co-occurring depression and chronic diseases often exhibit accelerated cognitive decline, poorer episodic memory, and reduced life satisfaction (5). These findings suggest that the combined burden of physical and mental health conditions may be greater than the sum of their individual effects.

Hypertension and diabetes, two highly prevalent chronic conditions, frequently co-occur with depression (2, 5). The coexistence of these conditions has been shown to lead to poorer disease management, slower recovery, and worse health outcomes. In such patients, the management of chronic diseases must be integrated with the management of mental health problems to enhance rehabilitation effects and improve quality of life. Especially in older adult populations with multiple comorbidities, simple physical rehabilitation interventions are often insufficient, making multidisciplinary rehabilitation strategies that integrate mental health support crucial for effective recovery (5).

While extensive research has been conducted on depression’s direct effects on individual chronic conditions, the interactive impact of depression and chronic diseases on broader health domains remains insufficiently explored. Specifically, there is limited understanding of the potential mediating role that conditions like hypertension might play in the relationship between depression and important health outcomes such as cognitive function, life satisfaction, and episodic memory (10). This knowledge gap impedes the development of effective integrated interventions.

The present study aims to address this research gap by examining not only the direct relationships between depression and various chronic diseases but also investigating whether the coexistence of depression and chronic diseases, particularly hypertension and diabetes, intensifies cognitive decline, diminishes life satisfaction, and impairs episodic memory. Furthermore, we explore the potential mediating role of blood pressure in these relationships, which could provide new insights into underlying physiological mechanisms. This study tests three primary hypotheses: (1) depression severity demonstrates varying relationships with different chronic disease risks; (2) the coexistence of depression and chronic diseases (hypertension and diabetes) exerts a synergistic negative effect on cognitive function, life satisfaction, and episodic memory that exceeds their individual effects; and (3) hypertension modifies the relationship between depression and cognitive outcomes through interactive effects in the studied population.

It should be noted that while this study examines depression’s association with heart disease and stroke as primary outcomes, hypertension and diabetes serve as the focus for interaction analyses, given their high prevalence and established relationships with both depression and cardiovascular outcomes in the Chinese population. This approach allows for examination of both direct disease outcomes and the chronic conditions that may mediate these relationships.

This multidimensional analysis aims to provide empirical evidence for integrated management strategies that simultaneously address mental health and chronic disease, thereby enhancing rehabilitation outcomes. The findings could inform the development of more holistic treatment approaches that consider both psychological and physiological factors in disease management. It is worth noting that all samples in this study were drawn from the China Health and Retirement Longitudinal Study (CHARLS) (11); thus, the population involved is primarily composed of Chinese individuals. As a result, the findings may not be broadly generalizable to other populations and should be interpreted within the context of the Chinese healthcare and sociocultural environment.

## Methods

### Study design and setting

This was a retrospective cohort study using data from the China Health and Retirement Longitudinal Study (CHARLS) collected between 2011 and 2020. The study specifically utilized data from two collection waves: the baseline survey in 2011 (wave 1) and the follow-up survey in 2020 (wave 5), creating a 9-year follow-up period to examine the relationships between depression, chronic diseases, and various health outcomes.

### Study population

By collecting comprehensive microdata on the Chinese population aged 45 and above, the CHARLS project aims to explore aging trends and promote the advancement of interdisciplinary research (11). The national baseline survey began in 2011 and used the PPS method of multi-stage probability sampling and proportional stratification to cover about 10,000 households and 17,000 participants in 450 villages from 150 counties in 28 provinces. CHARLS is a biennial, longitudinal survey. The study conducts in-home, face-to-face interviews with participants, making use of computer-assisted personal interviewing (CAPI) methods. The content covers basic information of individuals and households, financial support among family members, health, medical insurance, employment, income, expenditure, and assets. In addition, the survey incorporated 13 types of physical measurements and also involved the collection of blood samples. So far, CHARLS has completed five rounds of surveys: baseline survey (2011) and four follow-up visits (2013, 2015, 2018, 2020). CHARLS details refer to the previous literatures, data sets available in CHARLS website to download.^1^ Approved by the Biomedical Ethics Committee of Peking University, this project ensured that all participants provided written consent after receiving necessary information. This nationally representative longitudinal study employed a multi-stage probability sampling method with proportional stratification to ensure adequate representation of the Chinese population aged 45 and above.

**Figure 1:**
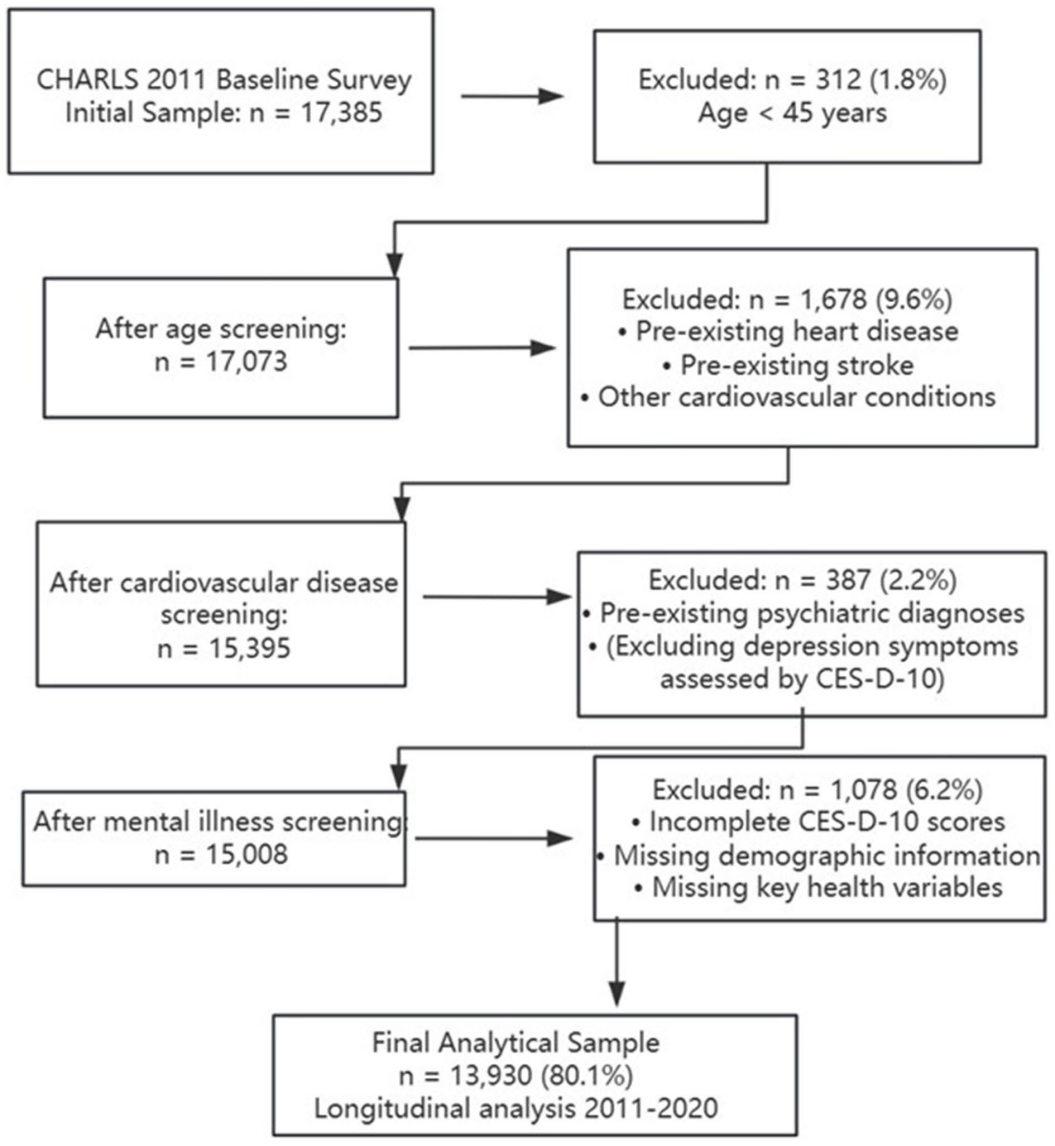
Participant selection flow chart.

The study made use of data from two collection waves: one in 2011 (wave 1) and another in 2020 (wave 5). The initial sample size was 17,385, and 3,455 participants were excluded after systematic screening. The exclusion criteria included: (1) age less than 45 years old, (2) diagnosed with cardiovascular disease at baseline survey in 2011, (3) diagnosed with mental illness at baseline survey in 2011, and (4) lack of other key information. In the final analysis, data from 13,930 participants were included. The systematic participant selection process followed sequential exclusion criteria to ensure data quality and temporal clarity (Figure 1). Participants were excluded based on: (1) age <45 years; (2) pre-existing cardiovascular disease at baseline; (3) pre-existing psychiatric diagnoses (excluding depression symptoms assessed by CES-D-10); and (4) incomplete key data. The final analytical sample included 13,930 participants (80.1% of initial sample) for longitudinal analysis spanning from 2011 to 2020.

### Data

The CES-D-10 (Center for Epidemiologic Studies Depression Scale) was included in the CHARLS questionnaire for assessing symptoms of depression (12). The CES-D-10 has proven to be a reliable and valid measure for detecting depressive symptoms, with particular applicability to middle-aged and older populations. Studies involving Chinese adults reveal that the CES-D-10 yields high sensitivity (0.85) and specificity (0.80) in this population. This 10-item scale, designed to be concise, asks participants to report how frequently they felt certain emotions, with options for responses: Four categories were used to classify timeframes: “little or no time (<1 day),” “some or very little time (1–2 days),” “occasionally or moderately often (3–4 days),” and “most of the time (5–7 days).” Responses were scored between 0 and 3, with higher total scores reflecting more severe depressive symptoms. A score of 10 or less was interpreted as “no depressive symptoms” (0 points), while a score greater than 10 indicated “depressive symptoms” (1 point). The overall scores were divided into four groups based on established cut-points and quartile distribution in similar populations: Group 1 (0–5, minimal symptoms), Group 2 (6–10, mild symptoms), Group 3 (11–16, moderate symptoms), and Group 4 (17–30, severe symptoms). This categorization allows for examination of dose–response relationships while maintaining adequate sample sizes in each group for statistical analysis.

Mental illness in this study was defined based on participants’ self-reported physician diagnoses of psychiatric or psychological conditions, including but not limited to anxiety disorders, bipolar disorder, schizophrenia, and other mood disorders, excluding depression which was assessed separately using the CES-D-10 scale. This definition was used consistently for both baseline exclusion criteria and outcome assessment during follow-up. It should be clarified that “mental health status” in the baseline characteristics analysis (Table 1) refers to current depressive symptoms measured by CES-D-10 scores (0 = no depressive symptoms, CES-D-10 ≤ 10; 1 = depressive symptoms, CES-D-10 > 10), which is distinct from pre-existing mental illness diagnoses that were excluded at baseline to ensure temporal clarity in our analysis. The specific mental illness assessment asked participants: “Have you been diagnosed by a psychiatrist with conditions such as anxiety disorders, depression (other than current symptoms assessed by CES-D-10), bipolar disorder, schizophrenia, or other mental health conditions?” This approach ensured clear separation between current depressive symptoms and pre-existing psychiatric diagnoses.

**Table 1:** Baseline characteristics and difference analysis of participants.

Variables	Total (*n* = 12,804)	0 (*n* = 9,504)	1 (*n* = 3,300)	Statistic	*p*
DL, Mean ± SD	0.28 ± 0.86	0.15 ± 0.59	0.67 ± 1.29	*t* = −22.28	**<0.001**
Age, Mean ± SD	58.89 ± 9.57	58.33 ± 9.43	60.49 ± 9.81	*t* = −11.05	**<0.001**
Episodic memory, Mean ± SD	3.62 ± 1.69	3.79 ± 1.71	3.10 ± 1.52	*t* = 20.12	**<0.001**
Mental state, Mean ± SD	8.18 ± 2.64	8.52 ± 2.49	7.11 ± 2.80	*t* = 23.46	**<0.001**
Cognitive ability, Mean ± SD	12.05 ± 3.53	12.53 ± 3.39	10.49 ± 3.54	*t* = 24.87	**<0.001**
IADL, Mean ± SD	0.37 ± 0.93	0.22 ± 0.69	0.81 ± 1.33	*t* = −24.47	**<0.001**
Sex, n(%)				χ^2^ = 196.16	**<0.001**
0	6,567 (51.29)	4,528 (47.64)	2039 (61.79)		
1	6,237 (48.71)	4,976 (52.36)	1,261 (38.21)		
Matrimony, n(%)				χ^2^ = 167.33	**<0.001**
0	1,568 (12.25)	954 (10.04)	614 (18.61)		
1	11,236 (87.75)	8,550 (89.96)	2,686 (81.39)		
Hypertension, n(%)				χ^2^ = 10.20	**0.001**
0	9,964 (78.16)	7,467 (78.85)	2,497 (76.17)		
1	2,784 (21.84)	2003 (21.15)	781 (23.83)		
Diabetes, n(%)				χ^2^ = 3.81	0.051
0	12,085 (95.08)	8,994 (95.30)	3,091 (94.44)		
1	626 (4.92)	444 (4.70)	182 (5.56)		
Cancer, n(%)				χ^2^ = 0.98	0.322
0	12,661 (99.16)	9,407 (99.21)	3,254 (99.03)		
1	107 (0.84)	75 (0.79)	32 (0.97)		
Lung disease, n(%)				χ^2^ = 68.06	**<0.001**
0	11,670 (91.29)	8,779 (92.50)	2,891 (87.79)		
1	1,114 (8.71)	712 (7.50)	402 (12.21)		
Arthritis, n(%)				χ^2^ = 403.52	**<0.001**
0	8,818 (68.94)	7,006 (73.79)	1812 (54.99)		
1	3,972 (31.06)	2,489 (26.21)	1,483 (45.01)		
Dyslipemia, n(%)				χ^2^ = 0.69	0.408
0	11,596 (92.12)	8,605 (92.00)	2,991 (92.46)		
1	992 (7.88)	748 (8.00)	244 (7.54)		
Liver disease, n(%)				χ^2^ = 10.52	**0.001**
0	12,375 (97.02)	9,218 (97.31)	3,157 (96.19)		
1	380 (2.98)	255 (2.69)	125 (3.81)		
Kidney disease, n(%)				χ^2^ = 68.93	**<0.001**
0	12,165 (95.31)	9,119 (96.22)	3,046 (92.67)		
1	599 (4.69)	358 (3.78)	241 (7.33)		
Gastropathy, n(%)				χ^2^ = 207.96	**<0.001**
0	10,198 (79.73)	7,859 (82.75)	2,339 (71.03)		
1	2,592 (20.27)	1,638 (17.25)	954 (28.97)		
Asthma, n(%)				χ^2^ = 58.00	**<0.001**
0	12,284 (96.27)	9,189 (97.02)	3,095 (94.10)		
1	476 (3.73)	282 (2.98)	194 (5.90)		
Memory disorder, n(%)				χ^2^ = 29.88	**<0.001**
0	12,639 (98.87)	9,410 (99.17)	3,229 (98.00)		
1	145 (1.13)	79 (0.83)	66 (2.00)		
Drinking history, n(%)				χ^2^ = 84.95	**<0.001**
0	8,413 (65.71)	6,028 (63.43)	2,385 (72.27)		
1	4,390 (34.29)	3,475 (36.57)	915 (27.73)		
Smoking history, n(%)				χ^2^ = 37.40	**<0.001**
0	8,712 (68.06)	6,325 (66.57)	2,387 (72.33)		
1	4,089 (31.94)	3,176 (33.43)	913 (27.67)		
Educational level, n(%)				χ^2^ = 466.10	**<0.001**
1	5,879 (45.95)	3,886 (40.93)	1993 (60.39)		
2	2,683 (20.97)	2031 (21.39)	652 (19.76)		
3	2,659 (20.78)	2,178 (22.94)	481 (14.58)		
4	1,574 (12.30)	1,400 (14.74)	174 (5.27)		

Total analytical sample *n* = 13,930; Table presents *n* = 12,804 participants with complete data for all baseline variables shown. Missing data: *n* = 1,126 (8.1%). t, *t*-test, χ^2^, Chi-square test; SD, standard deviation; For categorical variables: 0 = No/Absent, 1 = Yes/Present; Mental health status: 0 = No depressive symptoms (CES-D-10 ≤ 10), 1 = Depressive symptoms (CES-D-10 > 10); This mental health status classification is distinct from pre-existing mental illness diagnoses which were excluded at baseline. Bold values indicate statistical significance at *p* < 0.05.

The outcome measures of the study were events of heart disease, stroke and mental illness. Assessments were based on standardized questions from previous studies that included, “Were you ever diagnosed by a doctor with heart attack, coronary heart disease, angina, congestive heart failure, or another heart condition?” Moreover, “Did a doctor ever mention that you were diagnosed with a stroke?” Participants reporting heart disease, stroke, or mental illness during the follow-up were considered to have had the corresponding event. Illness was recorded as the interval between the last interview and the date of the interview at which the event was reported.

The determination of chronic diseases was made by the investigator by asking the question: “Did a doctor ever tell you that you were diagnosed with.? “In this study, participants were asked about two categories of chronic diseases: hypertension and diabetes or high blood sugar. For each chronic condition, a score of “1″ was recorded if the respondent reported a confirmed illness; if no diagnosis was made, a score of “0″ was recorded.

The selection of covariates was informed by existing literature on depression and chronic disease outcomes, as well as theoretical frameworks linking demographic, lifestyle, and health-related factors to mental health and chronic disease risks (5, 11, 12). Variables such as smoking and alcohol use were included due to their established associations with cardiovascular health and depression. Although economic status (e.g., income, employment) and social support are relevant, they were excluded due to data limitations or concerns about multicollinearity. Information on chronic conditions and covariates was derived from the baseline survey conducted in 2011, whereas data related to depression assessments were obtained during follow-up surveys spanning 2011 to 2020.

The internal validity of the study was ensured through CHARLS’s standardized data collection protocols, validated measurement instruments (such as the CES-D-10 with high sensitivity and specificity in Chinese populations), and quality control procedures. The external validity and generalizability of findings were supported by the comprehensive sampling strategy covering 28 Chinese provinces, though results may be most applicable to middle-aged and older Chinese populations given the study’s demographic focus.

### Control of confounding variables

Potential confounding was addressed through several strategies. First, participants with pre-existing cardiovascular disease or mental illness at baseline were excluded to establish clearer temporal relationships. Second, comprehensive data on potential confounders based on literature review was collected. These included demographic factors (age, sex, marital status), lifestyle variables (smoking, alcohol use), and health-related measures. Third, multivariable regression models adjusted for these identified confounders. While certain variables like economic status and social support are relevant, they were excluded due to data limitations or concerns about multicollinearity.

### Statistical methods

Of the 13,930 participants included in the final analytical sample, 12,804 (91.9%) had complete baseline data for all variables presented in the descriptive analysis (Table 1). The remaining 1,126 participants (8.1%) had missing data for one or more baseline characteristics but retained sufficient data for the primary longitudinal analyses. Missing data patterns were examined and found to be primarily missing at random, with no systematic bias identified across key demographic or health variables.

For participants with complete baseline data (*n* = 12,804), we used t-tests and Chi-square tests to compare variables across mental health status groups, evaluating differences between those with and without depressive symptoms. Tests of difference for continuous variables. To evaluate differences between groups classified by mental health status (0 and 1), an Independent Sample t-test was conducted on continuous variables, including age, Activities of Daily Living (ADL), episodic memory, mental status, cognitive function, and Instrumental Activities of Daily Living (IADL). An Independent Sample t-test was utilized to examine whether the mean difference between the two groups reached statistical significance, assuming the conditions of normality and variance homogeneity were met. The test results presented mean values, standard deviations (SD), t-values for the two groups, along with their corresponding *p*-values. A statistically significant result was defined by a *p*-value less than 0.05, highlighting a significant difference in the continuous variable between the two groups. Tests of difference for categorical variables. Included among the categorical variables were gender (male, female), marital status (e.g., married, single), health conditions such as hypertension, diabetes, cancer, lung disease, arthritis, liver disease, kidney disease, stomach disease, asthma, and memory impairment, as well as alcohol use, smoking behaviors, and education levels. A Chi-square test was used to identify significant differences in the distribution of categorical variables according to mental health status. By analyzing observed versus expected frequencies, it determines if group differences in categorical variables are statistically significant. Test results are reported as chi-square statistics (χ^2^ values) with corresponding *p* values. A *p*-value below 0.05 was regarded as statistically significant, denoting a meaningful difference between the two groups in that categorical variable. Setting of significance levels. For the sake of maintaining precision, the study adopted a *p*-value of less than 0.05 as the statistical significance threshold. This indicates that continuous and categorical variables differ significantly across mental health states when the *p*-value is below 0.05.

Multivariable logistic regression models were utilized in the study to compute Odds Ratios (ORs), aiming to analyze how depression groups affect the risks of heart disease, mental disorders, and stroke. The depression groupings used included groups 1 to 4, with Group 1 serving as the reference Group. Models controlled for age, sex, and other possible confounders.

In subgroup analyses, linear regression models were used to examine the effects of depression and chronic diseases (hypertension, diabetes) on life satisfaction, episodic memory scores, and cognitive performance. In order to further explore the interaction between depression and chronic diseases, the interaction terms of depression with hypertension and diabetes were also introduced in this study. The effect of age and gender on each outcome variable was controlled in the model.

This observational study design allows for examination of associations between depression and chronic disease outcomes but does not permit causal inferences. The temporal relationship between depression assessment and outcome measurement, combined with potential unmeasured confounding variables, limits our ability to establish causality. All findings should be interpreted as associations rather than causal relationships. Several methodological decisions require clarification. The depression symptom grouping (Groups 1–4) was based on clinical relevance and distribution characteristics in the Chinese population, ensuring adequate statistical power while maintaining clinically meaningful distinctions. The exclusion of participants with baseline cardiovascular disease and mental illness, while necessary for establishing temporal relationships, may limit generalizability to populations with existing comorbidities. The reliance on self-reported physician diagnoses, consistent with large-scale epidemiological studies, may introduce measurement bias but reflects real-world clinical practice in the Chinese healthcare system. The 9-year follow-up period provides sufficient time for incident disease development while minimizing loss to follow-up.

## Results

### Basic characteristics and differences analysis

**Figure 2:**
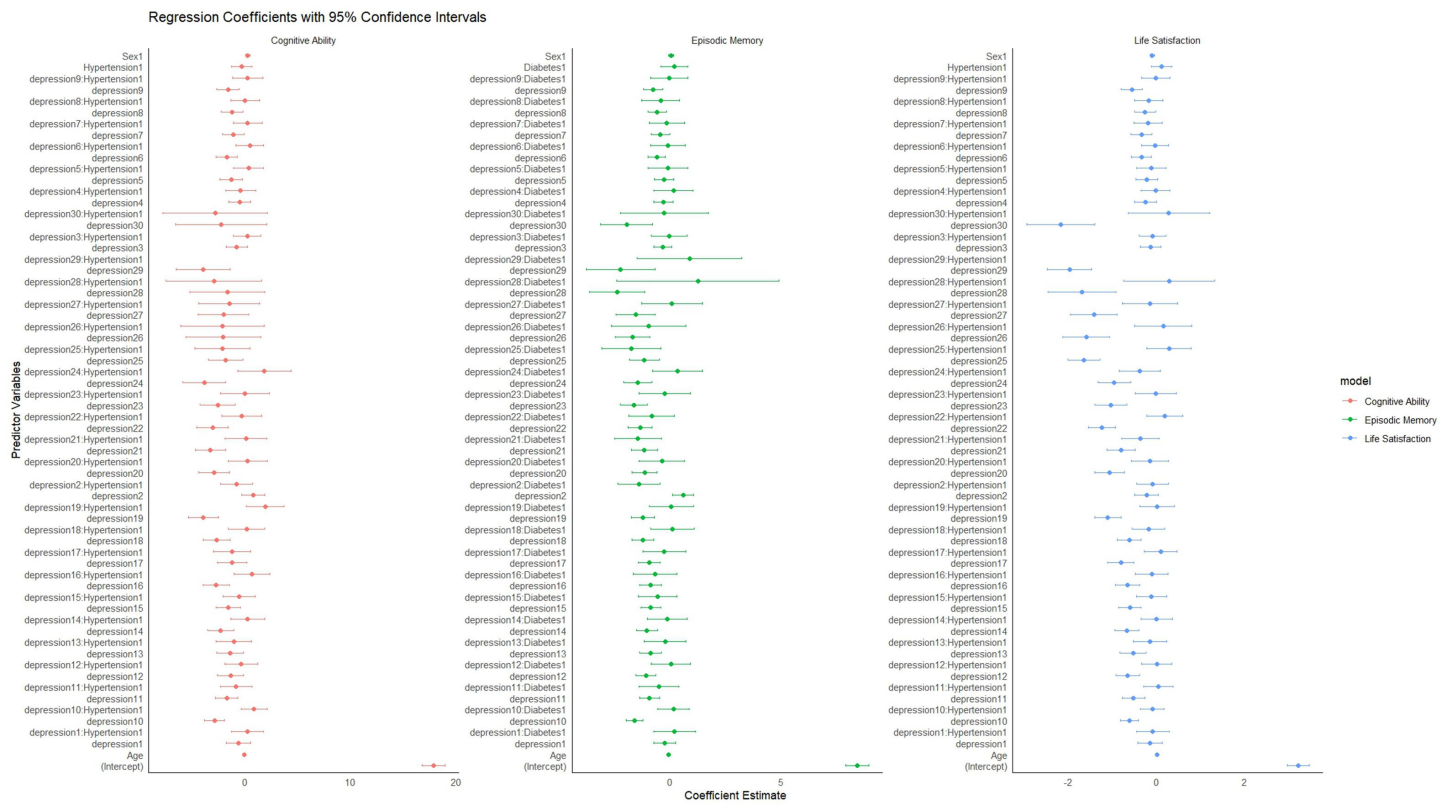
Forest plot of OR for heart disease, mental illness, and stroke across depression groups. Group 1 (CES-D-10: 0–5) serves as the reference category. Error bars represent 95% confidence intervals. OR >1 indicates increased risk; OR <1 indicates decreased risk compared to reference group. OR for heart disease, mental illness, and stroke, according to depression group.

Following systematic screening from the initial 17,385 CHARLS participants, a total of 13,930 participants (80.1%) were included in this study. For baseline characteristics analysis, 12,804 participants had complete data for all variables examined, among whom 9,504 participants (74.2%) were classified as having no depressive symptoms and 3,300 participants (25.8%) had depressive symptoms. An additional 1,126 participants (8.1%) had incomplete baseline data but were retained in the longitudinal analyses. Statistically significant differences were found in multiple variables between the two groups. Statistical significance (*p* < 0.05) was found for ADL, age, episodic memory, mental status, cognitive ability, IADL, gender, marital status, hypertension, lung disease, arthritis, liver disease, kidney disease, stomach disease, asthma, memory impairment, current drinking, smoking, and education. However, diabetes, cancer, and dyslipidemia were not statistically significant (*p* > 0.05; Table 1). As shown in Figure 2.

The effects of different depression groups on the outcomes of the three diseases were evaluated using OR and their corresponding 95% confidence intervals (CIs). The analysis revealed differential relationships: as depression severity increased, the risk for heart disease rose significantly, while showing an unexpected inverse relationship with stroke risk and a non-linear pattern with mental illness. Relative to the reference group (Group 1), individuals in the highest depression severity group (Group 4) experienced a 2.02-fold higher risk of heart disease, but contrary to expectations, showed a reduced risk of stroke (OR = 0.759) and no significant difference in mental illness risk (OR = 0.924, *p* = 0.513). These differential patterns, particularly the dose–response relationship between depression severity and heart disease risk, underline the necessity of early diagnosis and intervention for depressive symptoms while considering the complex relationship with other outcomes. The data reveal a clear positive association between depression severity and increased risk of heart disease. However, for stroke and mental illness, more complex patterns emerged. As depression levels rose across groups, heart disease risk escalated, while stroke risk actually decreased and mental illness showed a non-linear pattern. These findings emphasize the need for targeted monitoring and intervention approaches in clinical practice that consider these differential relationships.

Individual regression models for heart disease, stroke, and mental illness were developed using binary logistic regression models to examine the association between depression and different health outcomes. In the heart disease model, as depression severity increased from Group 1 to Group 4, individuals displayed a markedly higher relative risk of heart disease compared with the reference group, suggesting a strong relationship between depression and heart disease. In the stroke model, individuals with more severe depression displayed a reduced relative risk of stroke, potentially due to medical care or other underlying factors. In the psychiatric illness model: the relative risk of psychiatric illness was lower in individuals with more severe depression, particularly in Group 1 and Group 2. However, the difference between Group 4 and the reference group was not significant (Table 2). The three logistic regression models showed significant differences in the relationship between depression and different health outcomes (heart disease, stroke, mental illness). For heart disease, individuals with higher levels of depression had a significantly increased risk, indicating that depression is a strong risk factor for heart disease. For stroke, however, individuals with higher levels of depression instead showed a lower risk of stroke, which could be due to medical interventions or other underlying factors. For psychiatric disorders, the lower risk of psychiatric disorders in individuals with more severe depression, especially in the severe depression group, may be related to more frequent medical contacts. Overall, the associations of depression with different medical conditions show distinct patterns: a positive association with heart disease, an inverse relationship with stroke, and a complex non-linear pattern with mental illness. These differential relationships highlight the complex interplay between depression and various health outcomes, warranting further research to understand the underlying mechanisms.

**Table 2:** Estimated effects of different depression groups on the risk of heart disease, stroke, and mental illness.

Variable	Heart disease				Stroke				Mental illness			
	Estimate	SE	*p*	OR	Estimate	SE	*p*	OR	Estimate	SE	*p*	OR
Intercept	0.53948	0.06784	0.00001	1.715	−0.45123	0.06715	0.00001	0.637	−1.39705	0.08211	0.00001	0.247
depression_groupGroup1	0.88172	0.10553	0.00001	2.415	−0.77865	0.10177	0.00001	0.459	−1.34761	0.15742	0.00001	0.26
depression_groupGroup2	0.92373	0.10418	0.00001	2.518	−0.61707	0.09758	0.00001	0.539	−1.0827	0.14185	0.00001	0.339
depression_groupGroup3	0.86731	0.1094	0.00001	2.381	−0.60286	0.10288	0.00001	0.547	−0.72365	0.13753	0.00001	0.485
depression_groupGroup4	0.70341	0.10686	0.00001	2.02	−0.27588	0.09951	0.00557	0.759	−0.07904	0.1207	0.513	0.924

SE, standard errors; P, *p-*values; OR, odds ratios; Group 1 serves as the reference category. OR >1 indicate increased risk associated with higher depression severity levels.

### The impact of comorbid depression and chronic diseases on cognitive performance, life satisfaction, and episodic memory

Through linear regression models, this study systematically investigated the effects of depression and two widespread chronic conditions (hypertension and diabetes) on life satisfaction, episodic memory, and cognitive performance, along with exploring the interaction between depression and these conditions. The study results showed that depression significantly negatively influenced all three outcome variables, with the severity of depression amplifying the negative effects. The specific findings are as follows.

### The impact of depression level and hypertension on life satisfaction

**Figure 3:**
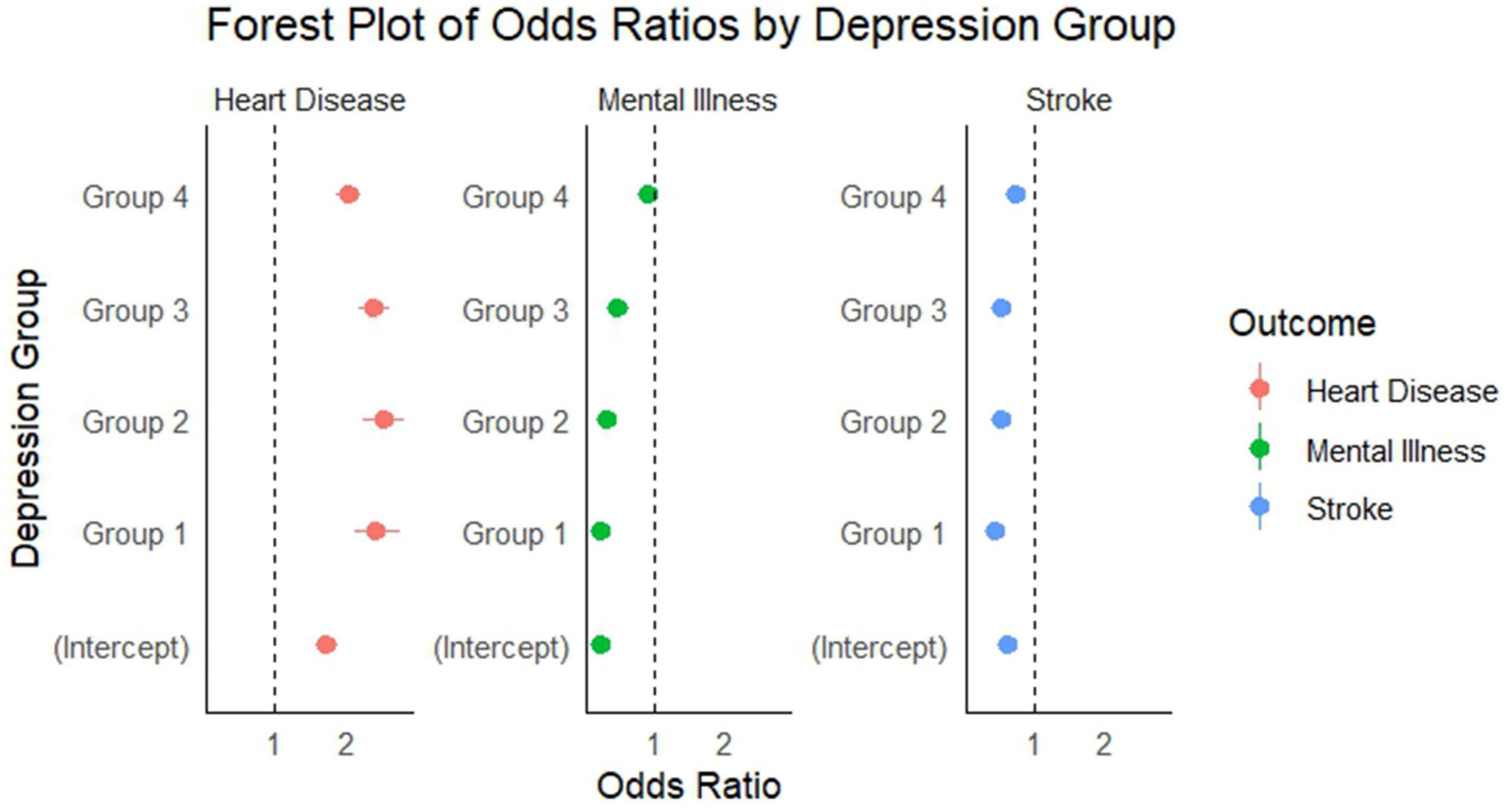
Regression coefficients of depression and chronic diseases on cognitive ability, episodic memory, and life satisfaction (95% confidence intervals). Points represent regression coefficients; horizontal lines represent 95% confidence intervals. Coefficients above zero indicate positive associations; below zero indicate negative associations.

Life satisfaction decreased significantly as depression scores increased. Individuals with high levels of depression had lower life satisfaction regardless of whether they had hypertension or not. However, individuals without hypertension had slightly higher median life satisfaction at each level of depression than individuals with hypertension, suggesting that hypertension may exacerbate the negative impact of depression on life satisfaction. This conclusion was further supported by regression analysis, in which depression scores (e.g., depression6, estimated value = −0.344, *p* = 0.0035 and depression9, estimated value = −0.561, *p* < 0.001) were significantly negatively associated with life satisfaction. While the direct effect of hypertension on life satisfaction was not statistically significant (*p* = 0.365), the analysis further showed that the interaction between depression and hypertension was also not significant (*p* > 0.05; Figure 3).

### The effect of depression level and diabetes on episodic memory

Depression had a significant negative effect on episodic memory, especially at high depression scores. The higher the depression score, the lower the episodic memory score. The significant interaction between depression and diabetes on episodic memory suggests a potential synergistic effect, where depression may exacerbate the cognitive impairments associated with diabetes. This phenomenon could be attributed to shared biological pathways, such as chronic systemic inflammation and neuroendocrine dysregulation, both of which play key roles in the pathophysiology of depression and diabetes. Additionally, the interaction may involve behavioral factors, including reduced treatment adherence and lifestyle changes in individuals affected by both conditions, which further contribute to cognitive decline. The findings point to the importance of implementing comprehensive management strategies that consider both mental health and chronic disease care (Figure 3).

### Effects of depression levels and hypertension on cognitive performance

Depression was found to significantly impair cognitive performance, with higher levels of depression associated with greater cognitive decline (e.g., depression6, estimate = −1.718, *p* = 0.0007 and depression10, estimate = −2.871, *p* < 0.001). Similar to life satisfaction and episodic memory, the interaction effect of depression and hypertension on cognitive performance was significant under some depression scores (e.g., depression19, estimated value = 1.927, *p* = 0.0348). This means that the negative effect of depression on cognitive performance is particularly prominent in patients with hypertension, possibly reflecting that hypertension somehow amplifies the effect of depression on brain cognitive function (Figure 3).

Overall, the regression analyses in this study showed that depression was a major risk factor for life satisfaction, episodic memory, and cognitive performance, and that its negative effects were more pronounced among individuals with chronic conditions, particularly hypertension and diabetes. Although the separate effects of hypertension and diabetes on life satisfaction, episodic memory, and cognitive performance were not significant, the interaction between hypertension and diabetes and depression had a significant negative effect on these health indicators in depressed individuals. Especially in episodic memory and cognitive ability, the double burden of chronic disease and depression can further aggravate functional impairment.

However, hypertension and diabetes alone did not have a significant effect on these outcome variables, suggesting that the effect of these chronic diseases was mainly reflected in their interaction with depression. This finding points to the critical role of depression screening and timely intervention in chronic disease patients to promote better quality of life and cognitive abilities.

## Discussion

The present study makes several novel contributions: (1) it is among the first to examine dose–response relationships between depression severity and multiple chronic disease outcomes in a Chinese population; (2) it quantifies the interactive effects of depression and chronic diseases on cognitive outcomes; and (3) it provides empirical evidence for the differential relationships between depression and various health conditions, challenging the assumption of uniform associations across all health outcomes.

The most important finding of this study is the identification of a clear dose–response association between depression severity and increased risk of heart disease, with individuals in the highest depression severity group experiencing approximately twice the risk compared to those with minimal depressive symptoms. Interestingly, our analysis revealed more complex, non-linear relationships between depression severity and the risks of stroke and mental illness. Furthermore, our results demonstrate significant interactive effects between depression and chronic diseases (particularly hypertension and diabetes) on cognitive function, life satisfaction, and episodic memory, suggesting a synergistic negative impact that exceeds the sum of their individual effects. These findings highlight the critical importance of integrating mental health screening and management into chronic disease care to enhance rehabilitation outcomes and quality of life.

### Relationship between depression and heart disease

Our findings revealed a strong positive association between depression severity and increased risk of heart disease, with OR ranging from 2.02 to 2.52 across depression groups compared to the reference group. This association is consistent with existing studies that have documented a similar relationship between depression and adverse cardiovascular outcomes (3, 13). The mechanisms underlying this association may involve both biological and behavioral pathways. From a biological perspective, depression is associated with dysregulation of the hypothalamic–pituitary–adrenal axis, increased inflammation, and altered autonomic nervous system function, all of which can contribute to the development and progression of cardiovascular disease (6, 9, 14, 15). Behaviorally, individuals with depression often exhibit poorer adherence to medication regimens, reduced physical activity, and unhealthy lifestyle behaviors such as smoking and poor dietary choices, which further increase cardiac risk (5).

However, our study differs from some previous findings in the magnitude of the association, potentially due to differences in population characteristics or methodology. In previous studies, depressed people had a 30 percent increased risk of coronary heart disease, our study found a much stronger association, with depressed individuals having over twice the risk (16). This discrepancy may be attributed to our focus on an older Chinese population and the use of different depression assessment tools, highlighting the importance of cultural and methodological considerations in interpreting these relationships.

### The relationship between depression and mental illness

Interestingly, our analysis of the relationship between depression and other mental illnesses revealed a complex non-linear pattern that contradicts the dose–response relationship observed with heart disease. While the highest depression severity group (Group 4) showed no significant difference in mental illness risk compared to the reference group (OR = 0.924, *p* = 0.513), lower depression severity groups (Groups 1–3) exhibited significantly reduced risks of mental illness. This model contrasts sharply with the existing research results, which generally document a strong comorbidity between depression and other mental illnesses. This discrepancy may be explained by several methodological factors specific to our study, including the use of retrospective self-reported data, our specific definition of mental illness, and potential cultural differences in diagnosing and reporting mental health conditions in the Chinese healthcare context.

Several explanations may account for this unexpected finding. First, individuals with more severe depression may receive earlier and more intensive mental health interventions, potentially reducing their risk of developing additional psychiatric conditions. Second, diagnostic overshadowing may occur, whereby clinicians attribute symptoms primarily to depression rather than recognizing comorbid conditions. Third, cultural factors influencing mental health reporting and help-seeking behaviors in the Chinese population could affect the observed relationship. For example, the stigma associated with mental illness in Chinese culture may lead to underreporting of psychiatric symptoms (17). These findings underscore the need for comprehensive psychiatric assessment in depressed individuals, particularly in cultural contexts where mental health stigma remains prevalent.

### The relationship between depression and stroke

Our analysis revealed an unexpected inverse relationship between depression severity and stroke risk, with depression groups showing lower OR (ranging from 0.459 to 0.759) compared to the reference group. This finding diverges from the dose–response relationship seen with heart disease and from the prevailing literature, which generally reports positive associations between depression and stroke risk (18). It is crucial to emphasize that this inverse pattern should not be interpreted as evidence that depression protects against stroke. Instead, this unexpected finding likely reflects methodological limitations, including: (1) potential survival bias where individuals with both severe depression and stroke may have higher mortality rates and thus be underrepresented in our sample; (2) differential healthcare utilization patterns among depressed individuals; (3) temporal ambiguity in the depression-stroke relationship due to the retrospective design; and (4) unmeasured confounding factors that require investigation in future prospective studies.

Several factors may explain this discrepancy. First, depressed individuals in our cohort may have received more intensive medical care and monitoring, potentially leading to better management of stroke risk factors. Second, the inverse relationship might reflect survival bias, as severely depressed individuals with stroke may have higher mortality rates and thus be underrepresented in our sample. Third, our study’s retrospective design may have introduced temporal ambiguity in the depression-stroke relationship.

Another possible explanation involves the role of antidepressant medications, which some studies suggest may have protective effects against certain types of strokes through anti-inflammatory or antiplatelet mechanisms (19). However, our study did not collect detailed information on medication use, limiting our ability to evaluate this hypothesis. Future prospective studies with comprehensive medication data are needed to clarify these complex relationships.

### The mediating role of blood pressure in the relationship between depression and cognitive function

Our study demonstrated that depression significantly impaired cognitive performance, with higher depression levels associated with greater cognitive decline. Notably, the interaction between depression and hypertension on cognitive performance was significant at certain depression levels (e.g., depression19, estimate = 1.927, *p* = 0.0348), suggesting that hypertension may amplify the negative effects of depression on cognitive function. This finding is consistent with existing research suggesting that high blood pressure is a key factor in cognitive decline (20, 21). The potential mechanisms underlying this interaction include cerebrovascular changes, white matter lesions, and altered cerebral blood flow regulation, which may be exacerbated in the presence of both depression and hypertension (12). Depression may influence cognitive function through mechanisms including increased cortisol levels, inflammatory processes, and reduced brain-derived neurotrophic factor (BDNF) (22). When combined with the vascular pathology associated with hypertension, these mechanisms may create a synergistic negative effect on cognitive performance. Our findings extend previous research by quantifying this interaction and highlighting the importance of considering both conditions in cognitive assessment and intervention. The clinical implications are significant: cognitive screening and intervention may be particularly important for patients with comorbid depression and hypertension, and treatment approaches targeting both conditions simultaneously may yield better cognitive outcomes than addressing either condition alone.

### The mediating role of blood pressure in the relationship between depression and life satisfaction

Our analysis showed that depression significantly reduced life satisfaction, with higher depression scores consistently associated with lower satisfaction levels. While the direct effect of hypertension on life satisfaction was not statistically significant (*p* = 0.365), individuals with hypertension had slightly lower median life satisfaction scores at each depression level compared to those without hypertension, suggesting a potential compounding effect. However, our study did not detect a significant independent effect of hypertension on life satisfaction. The absence of a significant interaction between depression and hypertension on life satisfaction in our study suggests that while both factors may influence life satisfaction, they likely operate through distinct pathways rather than a synergistic mechanism. Depression likely affects life satisfaction through psychological mechanisms such as negative cognitive biases and anhedonia, while hypertension may influence wellbeing through symptom burden, medication side effects, or awareness of having a chronic condition (5, 23). These findings highlight the importance of addressing depression as a primary target for improving life satisfaction in individuals with chronic conditions, as the psychological impact of depression appears to outweigh the direct effects of hypertension on subjective wellbeing in our study population.

### The mediating role of blood pressure in the relationship between depression and episodic memory

Our results demonstrated that depression had a significant negative effect on episodic memory, with higher depression scores associated with poorer memory performance. Interestingly, while we did not find a significant interaction between depression and hypertension on episodic memory, we observed a significant interaction between depression and diabetes, suggesting a potential synergistic effect specific to this combination. This finding has been replicated in other studies that have found additional cognitive effects of depression and diabetes (2, 24). The mechanisms underlying this interaction may involve shared pathophysiological pathways, including chronic inflammation, oxidative stress, and microvascular dysfunction, which are common to both conditions and can adversely affect hippocampal function and memory processes (13, 22, 25). The specificity of this interaction to diabetes rather than hypertension may reflect the distinct neurobiological effects of diabetes on memory systems. While hypertension primarily affects vascular health and white matter integrity, diabetes has been associated with specific disruptions in glucose metabolism in memory-critical brain regions and altered insulin signaling pathways that play important roles in synaptic plasticity and memory formation (26). These findings underscore the importance of targeted screening and intervention approaches for episodic memory deficits in individuals with comorbid depression and diabetes, as this population may be particularly vulnerable to accelerated memory decline and subsequent functional impairments.

### Implications for rehabilitation practice

Our findings provide evidence-based support for integrated rehabilitation approaches. The significant interactions between depression and chronic diseases on cognitive outcomes suggest that traditional rehabilitation focusing solely on physical conditions may be insufficient. Specifically, our data indicate that individuals with comorbid depression and hypertension or diabetes experience greater cognitive decline (as evidenced by the significant interaction effects shown in Table 2), suggesting these patients may require longer rehabilitation periods and more intensive interventions.

The dose–response relationship between depression severity and heart disease risk (ORs ranging from 2.02 to 2.52) provides quantitative evidence for the importance of mental health screening in cardiac rehabilitation programs. Furthermore, the differential effects observed across various health outcomes underscore the need for individualized rehabilitation strategies that consider both the severity of depression and the specific chronic conditions present.

These findings support the integration of psychological interventions, cognitive training, and traditional physical rehabilitation as evidenced by our quantified interactive effects on life satisfaction, cognitive function, and episodic memory.

### Policy brief for application by policymakers

The findings of this study have several important implications for health policy and healthcare system organization: 1. Integrated Care Models: Policymakers should prioritize the development and implementation of integrated care models that address both mental and physical health needs simultaneously. This could involve co-locating mental health services within primary care settings, implementing collaborative care models, and ensuring seamless communication between mental health and physical health providers. 2. Screening Protocols: Routine depression screening should be incorporated into the standard care for patients with chronic conditions, particularly those with hypertension and diabetes. Early detection and intervention for depression in these populations could significantly improve health outcomes and reduce healthcare costs. 3. Healthcare Professional Training: Investment in training programs for healthcare professionals to recognize and address the interactions between mental health and chronic diseases is essential. This includes training primary care physicians in basic mental health assessment and intervention, as well as educating mental health professionals about the management of common chronic conditions. 4. Insurance Coverage: Health insurance policies should provide adequate coverage for mental health services, particularly for individuals with chronic physical conditions. Reducing financial barriers to mental health care is crucial for implementing effective integrated care approaches. 5. Community-Based Programs: Support for community-based programs that address both mental and physical health, such as group-based interventions combining physical activity, chronic disease self-management, and psychological support, should be expanded. 6. Public Awareness Campaigns: Educational campaigns to reduce stigma surrounding mental health issues and increase awareness of the connections between mental and physical health would encourage earlier help-seeking behaviors and improve treatment adherence. Implementation of these policy recommendations could significantly improve the management of comorbid depression and chronic diseases, ultimately enhancing population health outcomes and reducing the economic burden associated with these conditions.

### Limitations

This study has several important limitations that must be considered when interpreting results. Most critically, the observational design precludes any causal inferences; all findings represent associations rather than causal relationships between depression and health outcomes. First, our reliance on self-reported data for depression and chronic diseases may introduce recall bias. Second, the CHARLS database focuses on middle-aged and older Chinese adults, limiting generalizability to other populations. Third, despite controlling for key variables, some potential confounders were not included. Fourth, the observational design precludes definitive causal inferences. Finally, our binary categorization of hypertension may have obscured more nuanced relationships between blood pressure, depression, and outcomes. Additionally, 8.1% of participants had incomplete baseline data, which may affect the generalizability of baseline characteristics, though sensitivity analyses confirmed no systematic bias in missing data patterns. Additionally, the unexpected inverse relationships observed for stroke and mental illness outcomes may reflect study-specific limitations including: (1) potential survival bias where individuals with both severe depression and stroke may have higher mortality rates; (2) differential healthcare access and monitoring patterns in depressed individuals; (3) cultural factors affecting mental health reporting and help-seeking behaviors in Chinese populations; and (4) the retrospective design introducing temporal ambiguity in depression-outcome relationships. These limitations underscore the need for prospective studies with repeated depression assessments and comprehensive outcome tracking.

## Conclusion

The results of this study indicate that depression severity has differential relationships with various health outcomes: a clear dose–response relationship with increased risk of heart disease, but more complex patterns with stroke and mental illness. Furthermore, depression interacts with chronic conditions such as hypertension and diabetes to exacerbate negative effects on cognitive function, life satisfaction, and episodic memory.

Based on the observed associations, mental health management may be beneficial as a core component of rehabilitation strategies to potentially improve patient recovery outcomes and quality of life, particularly for individuals with chronic diseases. A comprehensive approach combining cognitive training, chronic disease management, and psychological support is likely to yield the most beneficial outcomes.

Future research should focus on prospective studies with repeated measures to establish causal pathways, investigation of biological markers to elucidate mechanistic links, testing integrated management approaches, and exploring protective factors that may buffer negative effects of depression and chronic diseases. By addressing these research gaps and implementing integrated care models, healthcare providers and policymakers can develop more effective strategies to improve health outcomes for this vulnerable population. Specifically, future studies should: (1) employ prospective designs with multiple depression assessments over time to establish temporal relationships; (2) include biomarker data (inflammatory markers, cortisol, BDNF) to elucidate biological mechanisms; (3) investigate the unexpected inverse relationships with stroke and mental illness through larger, more diverse samples with comprehensive clinical validation; (4) examine the role of antidepressant medications and healthcare utilization patterns; (5) validate findings across different cultural and healthcare contexts; and (6) develop and test integrated intervention models that simultaneously address mental health and chronic disease management.

## Data Availability

The datasets presented in this study can be found in online repositories. The names of the repository/repositories and accession number(s) can be found at: The data that support the findings of this study are openly available in China Health and Retirement Longitudinal Study at http://charls.pku.edu.cn/index.html, reference number [N/A].

## Footnotes

^1^ http://charls.pku.edu.cn/en

## References

[1] Alexopoulos GS. Depression in the elderly. Lancet. (2005) 365:1961–70. doi: 10.1016/S0140-6736(05)66665-2 15936426

[2] Maina JG Balkhiyarova Z Nouwen A Pupko I Ulrich A Boissel M. Bidirectional Mendelian randomization and multiphenotype Gwas show causality and shared pathophysiology between depression and type 2 diabetes. Diabetes Care. (2023) 46:1707–14. doi: 10.2337/dc22-2373, PMID: 37494602 PMC10465984

[3] Whooley MA. Depression and cardiovascular disease. JAMA. (2006) 295:2874–81. doi: 10.1001/jama.295.24.2874, PMID: 16804154 PMC2771193

[4] Tang T Jiang J Tang X. Prevalence of depressive symptoms among older adults in mainland China: a systematic review and meta-analysis. J Affect Disord. (2021) 293:379–90. doi: 10.1016/j.jad.2021.06.050, PMID: 34246000

[5] Zhou P Wang S Yan Y Lu Q Pei J Guo W. Association between chronic diseases and depression in the middle-aged and older adult Chinese population—a seven-year follow-up study based on Charls. Front Public Health. (2023) 11:1176669. doi: 10.3389/fpubh.2023.1176669, PMID: 37546300 PMC10403076

[6] Dudek KA Dion-Albert L Lebel M LeClair K Labrecque S Tuck E. Molecular adaptations of the blood–brain barrier promote stress resilience vs. depression. Proc Natl Acad Sci USA. (2020) 117:3326–36. doi: 10.1073/pnas.1914655117, PMID: 31974313 PMC7022213

[7] Beurel E Toups M Nemeroff CB. The bidirectional relationship of depression and inflammation: double trouble. Neuron. (2020) 107:234–56. doi: 10.1016/j.neuron.2020.06.002, PMID: 32553197 PMC7381373

[8] Figee M Riva-Posse P Choi KS Bederson L Mayberg HS Kopell BH. Deep brain stimulation for depression. Neurotherapeutics. (2022) 19:1229–45. doi: 10.1007/s13311-022-01270-3, PMID: 35817944 PMC9587188

[9] Wang H He Y Sun Z Ren S Liu M Wang G. Microglia in depression: an overview of microglia in the pathogenesis and treatment of depression. J Neuroinflammation. (2022) 19:132. doi: 10.1186/s12974-022-02492-0, PMID: 35668399 PMC9168645

[10] Cai Y Chen M Zhai W Wang C. Interaction between trouble sleeping and depression on hypertension in the NHANES 2005–2018. BMC Public Health. (2022) 22.:481. doi: 10.1186/s12889-022-12942-2, PMID: 35277151 PMC8917766

[11] Hu Y Peng W Ren R Wang Y Wang G. Sarcopenia and mild cognitive impairment among elderly adults: the first longitudinal evidence from Charls. J Cachexia Sarcopenia Muscle. (2022) 13:2944–52. doi: 10.1002/jcsm.13081, PMID: 36058563 PMC9745544

[12] Qiu W Cai A Li L Feng Y. Association of depression trajectories and subsequent hypertension and cardiovascular disease: findings from the Charls cohort. J Hypertens. (2024) 42:432–40. doi: 10.1097/HJH.0000000000003609, PMID: 37937504

[13] Zhou W Sun L Zeng L Wan L. Mediation of the association between sleep disorders and cardiovascular disease by depressive symptoms: an analysis of the National Health and nutrition examination survey (Nhanes) 2017–2020. Prev Med Rep. (2023) 33:102183. doi: 10.1016/j.pmedr.2023.102183, PMID: 37223583 PMC10201828

[14] Liu L Wang H Chen X Zhang Y Zhang H Xie P. Gut microbiota and its metabolites in depression: from pathogenesis to treatment. EBioMedicine. (2023) 90:104527. doi: 10.1016/j.ebiom.2023.104527, PMID: 36963238 PMC10051028

[15] Tobaldini E Carandina A Toschi-Dias E Erba L Furlan L Sgoifo A. Depression and cardiovascular autonomic control: a matter of vagus and sex paradox. Neurosci Biobehav Rev. (2020) 116:154–61. doi: 10.1016/j.neubiorev.2020.06.029, PMID: 32598983

[16] Ski CF Taylor RS Mcguigan K. Psychological interventions for depression and anxiety in patients with coronary heart disease, heart failure or atrial fibrillation. Cochrane Database Syst Rev. (2024) 4:CD013508. doi: 10.1002/14651858.CD013508.pub3 PMC10996021 38577875

[17] Boyd JE Adler EP Otilingam PG Peters T. Internalized stigma of mental illness (Ismi) scale: a multinational review. Compr Psychiatry. (2014) 55:221–31. doi: 10.1016/j.comppsych.2013.06.005, PMID: 24060237

[18] Villa RF Ferrari F Moretti A. Post-stroke depression: mechanisms and pharmacological treatment. Pharmacol Ther. (2018) 184:131–44. doi: 10.1016/j.pharmthera.2017.11.005, PMID: 29128343

[19] Frank D Gruenbaum BF Zlotnik A Semyonov M Frenkel A Boyko M. Pathophysiology and current drug treatments for post-stroke depression: a review. Int J Mol Sci. (2022) 23:15114. doi: 10.3390/ijms232315114, PMID: 36499434 PMC9738261

[20] Contreras-Osorio F Ramirez-Campillo R Cerda-Vega E Campos-Jara R Martínez-Salazar C Reigal RE. Effects of physical exercise on executive function in adults with depression: a systematic review and meta-analysis. Int J Environ Res Public Health. (2022) 19:15270. doi: 10.3390/ijerph192215270, PMID: 36429985 PMC9690406

[21] Marwaha S Palmer E Suppes T Cons E Young AH Upthegrove R. Novel and emerging treatments for major depression. Lancet. (2023) 401:141–53. doi: 10.1016/S0140-6736(22)02080-3, PMID: 36535295

[22] Colucci-D’amato L Speranza L Volpicelli F. Neurotrophic factor Bdnf, physiological functions and therapeutic potential in depression, neurodegeneration and brain cancer. Int J Mol Sci. (2020) 21:7777. doi: 10.3390/ijms21207777 PMC7589016 33096634

[23] Ooi PB Khor KS Tan CC Ong DLT. Depression, anxiety, stress, and satisfaction with life: moderating role of interpersonal needs among university students. Front Public Health. (2022) 10:958884. doi: 10.3389/fpubh.2022.958884, PMID: 36249213 PMC9554619

[24] Li S Yang D Zhou X Chen L Liu L Lin R. Neurological and metabolic related pathophysiologies and treatment of comorbid diabetes with depression. CNS Neurosci Ther. (2023) 30:e14497. doi: 10.1111/cns.14497, PMID: 37927197 PMC11017426

[25] Dudek KA Dion-Albert L Kaufmann FN Tuck E Lebel M Menard C. Neurobiology of resilience in depression: immune and vascular insights from human and animal studies. Eur J Neurosci. (2019) 53:183–221. doi: 10.1111/ejn.14547, PMID: 31421056 PMC7891571

[26] Abbas Q Latif S Ayaz Habib H Shahzad S Sarwar U Shahzadi M. Cognitive behavior therapy for diabetes distress, depression, health anxiety, quality of life and treatment adherence among patients with type-ii diabetes mellitus: a randomized control trial. BMC Psychiatry. (2023) 23:86. doi: 10.1186/s12888-023-04546-w, PMID: 36737757 PMC9896442

